# Correlates of University Students’ Soft and Energy Drink Consumption According to Gender and Residency

**DOI:** 10.3390/nu7085298

**Published:** 2015-08-06

**Authors:** Tom Deliens, Peter Clarys, Ilse De Bourdeaudhuij, Benedicte Deforche

**Affiliations:** 1 Department of Human Biometry and Biomechanics, Vrije Universiteit Brussel, Pleinlaan 2, Brussels 1050, Belgium; E-Mails: pclarys@vub.ac.be (P.C.); Benedicte.Deforche@vub.ac.be (B.D.); 2 Department of Movement and Sports Sciences, Ghent University, Watersportlaan 2, Ghent 9000, Belgium; E-Mail: ilse.debourdeaudhuij@ugent.be

**Keywords:** determinants, sugar sweetened beverages, soda, caffeinated beverages, moderators, college

## Abstract

This study assessed personal and environmental correlates of Belgian university students’ soft and energy drink consumption and investigated whether these associations were moderated by gender or residency. Four hundred twenty-five university students completed a self-reported on-line questionnaire assessing socio-demographics, health status, soft and energy drink consumption, as well as personal and environmental factors related to soft and energy drink consumption. Multiple linear regression analyses were conducted. Students believing soft drink intake should be minimized (individual subjective norm), finding it less difficult to avoid soft drinks (perceived behavioral control), being convinced they could avoid soft drinks in different situations (self-efficacy), having family and friends who rarely consume soft drinks (modelling), and having stricter family rules about soft drink intake were less likely to consume soft drinks. Students showing stronger behavioral control, having stricter family rules about energy drink intake, and reporting lower energy drink availability were less likely to consume energy drinks. Gender and residency moderated several associations between psychosocial constructs and consumption. Future research should investigate whether interventions focusing on the above personal and environmental correlates can indeed improve university students’ beverage choices.

## 1. Introduction

Sugar sweetened beverage consumption (including the full range of soft drinks, fruit drinks, sports drinks) has increased considerably across the globe during the last decades [1,2]. Malik’s review [1] clearly showed that sugar sweetened beverage intake significantly contributes to weight gain and can lead to increased risk of type 2 diabetes and cardiovascular disease. Sugar sweetened beverages, typically containing sucrose, high-fructose corn syrup, or fruit juice concentrates, may lead to weight gain through the high added sugar content, low satiety, and potential incomplete compensation for total energy, leading to increased energy intake [1]. Today, it is also hypothesized that artificially sweetened beverage consumption may be related to weight gain, diabetes, and cardiovascular disease. Due to a dysregulation of appetite control, as a consequence of the mismatch between the intense taste of sweetness during consumption and the lack of energy consumed, artificially sweetened beverages may increase weight [3]. Furthermore, studies have demonstrated an effect of artificial sweeteners and diet beverages on incretin hormones, which would influence insulin secretion and thus blood glucose control [3]. Accordingly, consumption of artificially sweetened beverages has been associated with an increased risk for diabetes [4].

Similar to soft drinks, the consumption of energy drinks has increased markedly during the last two decennia [5,6,7]. Along with the exponential growth of the energy drink market [5], there are also increasing concerns regarding the potential health effects of energy drink consumption [7]. Energy drinks (Red Bull, Nalu, Burn, Monster) are known to stimulate cognitive functioning and alertness and typically contain large doses of stimulants, such as caffeine (ranging from 50 to 505 mg per can or bottle), taurine, guarana, but also sucrose, B vitamins, sodium, and other minerals [2,5,8]. Sucrose and caffeine containing drinks are thought to play a role in the epidemic of obesity and type 2 diabetes [9,10]. Although caffeine generally increases fat oxidation [11] and may even have a positive effect on long-term weight management when combined with ephedrine [12], Rush and colleagues [13] showed that combining sucrose and caffeine (in the form of energy drinks) may increase carbohydrate oxidation and suppress fat oxidation. It was also suggested that energy drink consumption may even cause lipogenesis [13]. Furthermore, caffeine may act as an adenosine receptor antagonist and lowers glucose uptake, proposing that skeletal muscle becomes more resistant to insulin with the administration of caffeine [14].

Current recommendations are to limit total soft drink (sugar and artificially sweetened beverages) and energy drink consumption and replace them with healthy alternatives such as water [1,3].

Previous research has shown that going to college or university can be a critical period for unhealthy weight related behaviors including sweetened beverage consumption [15,16]. University and college students are heavy consumers of sugar sweetened beverages. Sixty-five percent of students reported daily consumption of some form of sugar sweetened beverages, with an estimated caloric intake of 543 ± 671 kCal/day [17]. Also energy drinks are very popular in this particular population. In the US, up to 59% of college students reported energy drink consumption during the last week, whereas 29% of students consumed one or more energy drinks the day before completing the survey [18]. Among Turkish students, 10.3% of current energy drink users reported daily intake [19]. Moreover, males were more likely than females to consume sugar sweetened beverages and energy drinks daily [17,18,20]. Although Brunt and colleagues [21] showed that beverage intake among college students may vary according to residency, however, to date, no studies have assessed the association between residency and soft and energy drink consumption. Previous studies mainly focused on consumption of sugar sweetened beverages alone, rather than total (sugar and artificially sweetened) soft drink consumption. To the best of our knowledge, no European data on energy drink consumption in university students exist.

To develop effective and tailored intervention strategies aiming to decrease university students’ soft and energy drink intake, it is important to get insight into factors associated with students’ consumption. Focus groups in six US colleges revealed that taste was the most important reason for choosing a non-alcoholic beverage, whereas price was the second most commonly mentioned factor influencing choice [22]. According to the same study, health and nutritional content of beverages had limited influence on choice [22]. Another focus group study in Australian university students reported that non-alcoholic beverage consumption was related to some social cues (setting in which alcohol is usually consumed, socializing with friends, and family influences), and some physical environmental cues (purchasing of fast foods, and ready availability, pricing and promotion of caloric beverages) [23]. Furthermore, other intrinsic qualities (such as sugar and caffeine content, and their association with treats and rewards) and personal health beliefs were also seen as important influences on consumption [23]. More specifically for caffeinated beverages, non-European literature showed that students may consume energy drinks to keep awake, to increase energy, to boost performance during exercise, to concentrate while studying, and to drink with alcohol while partying [19,20,24]. Apart from the abovementioned reasons or motivations to drink energy drinks, to date no correlates of energy drink consumption have been assessed from a multilevel (combining personal with social and physical environmental factors) behavioral viewpoint.

To the best of our knowledge, only qualitative studies [22,23] assessing determinants or correlates of university students’ soft drink consumption have been conducted. Quantitative studies are needed in order to explain to what extent personal and environmental factors are associated with soft drink consumption among university students. Moreover, no European studies have assessed correlates of energy drink consumption in this population. Although studies have shown that university students’ weight related behaviors may vary according to gender and residency [17,21], to date, no studies have assessed whether personal and environmental correlates of soft drink consumption differ by gender or residency. Including gender and residency as possible moderators may potentially yield important information on whether specific subgroups of students should be approached differently when designing intervention strategies. Therefore, the first objective of this study was to assess which personal and environmental factors were related to Belgian university students’ soft drink and energy drink consumption. The second objective was to investigate whether these associations were moderated by gender or residency.

## 2. Experimental Section

### 2.1. Participants

A random sample of 816 university students (including students of all study years) was contacted face-to-face on the university campus. All contacted students orally consented to participate in the study and received an on-line questionnaire invitation by e-mail. Two reminder e-mails were sent to every student. Four hundred sixty-seven (57.2%) students completed the questionnaire, with 425 of them (52.1%) completing the questionnaire entirely.

### 2.2. Procedure

In this cross-sectional study, students were asked to complete a self-reported on-line questionnaire consisting of questions derived from existing questionnaires [25,26,27,28,29,30]. The aim was to assess socio-demographic variables, health status, soft and energy drink consumption (see Table 1), as well as personal and environmental factors related to soft and energy drink consumption (see Table 2).

**Table 1:** Sample characteristics and students’ beverage consumption (%, Mean ± SD).

*n* = 425	%, Mean ± SD
*Demographics*	
Gender (% females)	59.8
Age (years)	21.2 ± 2.1
Ethnicity (% of students of which one of the parents is from foreign origin)	29.6
Residency (% living in a student residence)	36.3
*Socio Economic Status (SES)*	
Education mother (% diploma higher education)	61.0
Education father (% diploma higher education)	61.7
*General health*	
BMI (kg/m^2^)	21.8 ± 2.9
Underweight (%)	9.5
Normal weight (%)	78.7
Overweight (%)	10.4
Obese (%)	1.4
Smoking (% non-smokers)	87.3
Perceived health (% reporting good to very good health)	75.0
Perceived fitness (% reporting good to very good fitness)	43.9
*Beverage consumption*	
Total soft drink consumption (mL/day)	423.6 ± 445.2
Sugar sweetened carbonated beverages (mL/day)	219.3 ± 352.4
Artificially sweetened carbonated beverages (mL/day)	75.9 ± 194.3
Orange juice (mL/day)	69.1 ± 110.7
Other juices (mL/day)	41.3 ± 92.1
Sports drinks (mL/day)	18.6 ± 44.8
Total energy drink consumption (mL/day)	19.9 ± 62.4
Sugar sweetened energy drinks (mL/day)	17.2 ± 59.0
Artificially sweetened energy drinks (mL/day)	2.7 ± 14.1
Water (mL/day)	649.9 ± 270.9

**Table 2:** Personal and environmental factors of soft and energy drink consumption (Mean ± SD; *n* = 425).

Variable name (number of items) ^#^	Content	Scoring	Soft drinks Mean ± SD	Energy drinks Mean ± SD
*Personal factors*				
Taste preference (1) ^a^	How tasty are soft/energy drinks to you?	0 = not tasty at all; 10 = very tasty	6.6 ± 1.9	4.3 ± 3.0
Attitude (1) ^b^	How do you feel about drinking soft/energy drinks? Drinking soft/energy drinks is: …	1 = very bad; 5 = very good	2.4 ± 0.7	1.9 ± 0.7
Individual subjective norm (1) ^c^	I believe I should avoid drinking soft/energy drinks on most days of the week	1 = strongly disagree; 5 = strongly agree	3.7 ± 1.2	4.5 ± 0.8
Perceived control (2) ^c^	How hard is it to avoid drinking soft/energy drinks at home/at university?	1 = very hard; 5 = not hard at all	3.7 ± 1.0	4.6 ± 0.8
Habit strength (3) ^d^	Drinking soft/energy drinks is something that I almost automatically do/I regularly do/typically me	1 = strongly disagree; 5 = strongly agree	2.5 ± 1.2	1.4 ± 0.8
Self-efficacy (11/13) ^c^	Confidence to avoid soft/energy drinks in potentially difficult situations (e.g. if you are going out, during exams)	1 = I know for sure I cannot; 5 = I know for sure I can	3.7 ± 0.8	4.4 ± 0.8
Perceived benefits (9/11) ^c^	Agreement with positive effects of avoiding soft/energy drinks (e.g. healthy, spending less money, no palpitations)	1 = strongly disagree; 5 = strongly agree	3.4 ± 0.7	3.5 ± 0.9
Perceived barriers (12/15) ^c^	Agreement with possible barriers to avoid drinking soft/energy drinks (e.g. lack of self-discipline, temptation)	1 = strongly disagree; 5 = strongly agree	2.0 ± 0.7	1.8 ± 0.8
*Environmental factors*				
Social norm (3) ^c^	Do(es) your partner/parents/friends believe you should avoid drinking soft/energy drinks on most days of the week?	1 = not at all; 5 = totally	2.8 ± 0.9	2.7 ± 1.3
Social support (3) ^c^	Do(es) your partner/parents/friends support you (or would support you if you would try) to avoid drinking soft/energy drinks on most days of the week?	1 = never; 5 = very often	3.0 ± 1.1	3.3 ± 1.3
Modelling (3) ^c^	How often do(es) your partner/parents/friends drink soft/energy drinks?	1 = never; 5 = daily	3.5 ± 0.9	2.0 ± 0.7
Family rules (2) ^b^	How often were you (earlier)/are you (now) allowed to drink soft/energy drinks at home?	1 = never; 5 = always	3.6 ± 1.0	2.2 ± 1.3
Perceived availability (5) ^b^	To what extent are soft/energy drinks available at home or student residence/in on-campus vending machines/in the student restaurant/in on-campus cafeterias/in campus surroundings?	1 = never; 5 = always	4.5 ± 0.5	3.2 ± 1.1
Distance to stores (2) ^a^	How far is it from home/student residence to the nearest place or store where you can buy soft/energy drinks?	1 = less than 50 m; 7 = more than 10 km	3.2 ± 1.3	3.2 ± 1.3

^#^ All variables were calculated by averaging the scores on the items included; ^a^ Bere *et al.* [27]; ^b^ Ezendam *et al.* [29]; ^c^ Vandelanotte *et al.* [26]; ^d^ Verplanken & Orbell [25].

### 2.3. Personal and Environmental Correlates

The questionnaire included questions based on factors derived from psychosocial models used to explain health behavior, namely, Theory of Planned Behavior [31] and Social Cognitive Theory [32] and was completed with psychosocial and physical environmental questions based on results of previous focus group research in university students [22,23,33]. In the current study, all items representing psychological constructs were categorized as “personal factors”, whereas all social and physical environmental items were categorized as ‘environmental factors’. More details on how every item was measured are presented in Table 2.

### 2.4. Beverage Consumption

Soft drink consumption was defined as consumption of sugar sweetened (Coca-Cola, Fanta, Atlanta, GA, USA) and artificially sweetened carbonated beverages (Coca-Cola light, Fanta light), orange and other fruit juices, and sports drinks. Energy drink consumption was defined as consumption of caffeinated sugar or artificially sweetened carbonated beverages (Red Bull (light), Salzburg, Austria, Nalu). To measure daily soft and energy drink consumption (in mL) participants were asked to answer the following question, derived from a validated food frequency questionnaire (FFQ) [28]: “How often do you consume the following beverages: (1) Coca-Cola, Fanta, Sprite, Ice Tea, or other sugar sweetened carbonated beverages; (2) Coca-Cola light/zero, Fanta light/zero, Sprite light/zero, Ice Tea light or other artificially sweetened carbonated beverages; (3) orange juice, fresh or non-fresh; (4) other juices; (5) Aquarius, AA Drink, or other sports drinks; (6) Red Bull, Nalu, Burn, Monster or other sugar sweetened energy drinks; (7) Red Bull light or other artificially sweetened energy drinks; (8) water (of any kind). For every item the following frequency categories were used: (1) never; (2) one to three times per month; (3) one time per week; (4) two to four times per week; (5) five to six times per week; (6) once a day; (7) two to three times per day; (8) four to six times per day; (9) more than six times per day. Missing frequencies were set to zero. To calculate beverage consumption, frequency (per day) was multiplied by a standard portion size (sugar and artificially sweetened carbonated beverages, and sports drinks = 330 mL; orange juice and other juices = 200 mL; energy drinks (regular and diet) = 250 mL; water = 150 mL) [34]. To calculate total soft drink consumption, sugar and artificially sweetened carbonated beverages, orange and other juices, and sports drinks were summed. To calculate total energy drink consumption, regular and diet energy drinks were summed.

### 2.5. Ethics Statement

All participants voluntarily completed an on-line questionnaire anonymously. The study was approved by the Medical Ethical Committee of the university hospital (Vrije Universiteit Brussel, Brussels, Belgium) on 5 October 2011 (B.U.N. 143201111941). All procedures followed were in accordance with the ethical standards of the responsible committee on human experimentation (institutional and national) and with the Helsinki Declaration of 1975, as revised in 2000.

### 2.6. Statistical Analyses

Data were analyzed using IBM SPSS Statistics 22. Independent samples *t*-tests and *chi*^2^-tests were used to compare basic characteristics of respondents excluded from the sample (students who did not complete the questionnaire entirely) with basic characteristics of the study sample. After checking for multicollinearity (*r* > 0.6), multiple linear regression analyses were conducted to determine factors related to soft and energy drink consumption. Firstly, a model that included the main effects of personal and environmental factors and potential moderators (gender and residency) was estimated. Secondly, different models were estimated which included the main effects and the interaction effect between the independent variables (personal and environmental factors) and one of the two moderators. Thirdly, a final model was built that combined the main effects with all significant interactions observed in the previous step. Significant interaction terms were explored according to established procedures [35]. Age, ethnicity, parental education, and body mass index (BMI) were included as covariates in all analyses. For main effects *p* < 0.05 were considered as statistically significant. As interaction effects have less power, significance was set at *p* < 0.1 to estimate significant interaction effects [36].

## 3. Results

### 3.1. Comparison of Excluded Respondents with the Study Sample

Basic characteristics of respondents excluded from the sample (students who did not complete the questionnaire entirely) were compared with basic characteristics of the study sample. The study sample (*n* = 425) and excluded respondents (*n* = 42) did not differ for gender (59.8 *vs.* 65.9% female respondents; *chi*^2^ = 0.6; *p* = 0.450), age (21.2 ± 2.1 *vs.* 21.8 ± 2.4 years; *t* = 1.6; *p* = 0.111) and BMI (21.8 ± 3.0 *vs.* 21.8 ± 2.9 kg/m^2^; *t* = −0.1; *p* = 0.933).

### 3.2. Sample Characteristics

The sample consisted of 59.8% female respondents with a mean age of 21.2 ± 2.1 years. About one-third resided in a student residence. Total soft drink consumption was 423.6 ± 445.2 mL/day, whereas total energy drink consumption was 19.9 ± 62.4 mL/day. Additional sample characteristics are presented in Table 1.

### 3.3. Personal and Environmental Correlates of Soft and Energy Drink Consumption

Table 2 gives an overview of potential personal and environmental correlates of soft and energy drink consumption in Belgian university students with reference to the original questionnaires.

Table 3 shows the multivariate regression model of personal and environmental correlates of soft drink consumption in Belgian university students. After checking for multicollinearity (showing *r* = −0.63 between perceived behavioral control and habit strength) habit strength was excluded from the multiple regression model. After controlling for age, ethnicity, parental education, and BMI, perceived behavioral control was negatively related to soft drink consumption; the more students found it difficult to avoid soft drinks, the higher their soft drink consumption. Self-efficacy towards avoiding soft drinks was negatively associated with soft drink intake; the more students were convinced they can avoid soft drinks in different situations, the lower students’ soft drink consumption. The main effects of individual subjective norm and modelling were moderated by residency, whereas the main effect of family rules was moderated by gender. Individual subjective norm towards avoiding soft drink consumption was negatively associated with soft drink consumption in students living at home (β = −0.345; *p* < 0.001); the more students living at home were convinced they should minimize their soft drink intake, the less these students drank soft drinks. No main effect of individual subjective norm was found in students living in a student residence (β = −0.156; *p* = 0.090). Modelling of soft drink consumption was positively associated with soft drink intake in students living at home (β = 0.186; *p* = 0.005), but not in students living in a student residence (β = −0.026; *p* = 0.781); the more students’ partner, parents and friends or student colleagues drank soft drinks, the higher students’ soft drink consumption. Family rules about soft drink intake was positively associated with soft drink consumption in male students (β = 0.234; *p* = 0.009); the more male students were/are allowed (by their parents) to drink soft drinks, the higher their soft drink intake. No main effect of family rules was detected in female students (β = 0.045; *p* = 0.556). Finally, education of the father (control variable) was also negatively related to soft drink consumption; the higher fathers’ educational level, the lower students’ soft drink consumption. The strongest correlate was individual subjective norm (β = −0.345; *p* < 0.001). The total model explained 44.7% of the variance in soft drink consumption.

**Table 3:** Multivariate regression model of personal and environmental correlates of soft drink consumption in Belgian university students (*t*-values, β-values, Adjusted *R*^2^).

*n* = 425	*t*	β	Adj *R*^2^
*Control variables*			
Age	−1.3	−0.058	
Ethnicity (0 = parents from Belgian origin; 1 = one of parents from foreign origin)	0.4	0.018	
Education mother (0 = no diploma higher education; 1 = diploma higher education)	−0.1	−0.006	
Education father (0 = no diploma higher education; 1 = diploma higher education)	−2.8 **	−0.129	
BMI	−1.5	−0.070	
*Moderators*			
Gender (0 = male; 1 = female)	1.3	0.220	
Residency (0 = living at home; 1 = living in a student residence)	0.1	0.027	
*Personal and environmental correlates*			
Taste preference	0.0	0.001	
Attitude	0.2	0.008	
Individual subjective norm	−4.8 ***	−0.345	
Perceived control	−2.2 *	−0.125	
Self-efficacy	−4.2 ***	−0.239	
Perceived benefits	−0.3	−0.017	
Perceived barriers	−0.8	−0.042	
Social norm	1.7 ^	0.077	
Social support	0.0	0.001	
Modelling	2.9 **	0.186	
Family rules	2.6 **	0.234	
Perceived availability	1.6	0.075	
Distance to stores	0.6	0.026	
*Moderation effects of gender and residency*			
Individual subjective norm x residency	1.7 ^	0.311	
Perceived benefits x residency	0.4	0.129	
Modelling x residency	−1.9 ^	−0.411	
Family rules x gender	−2.1 *	−0.345	
Family rules x residency	−0.6	−0.111	
			0.447

* *p* < 0.05, ** *p* < 0.01, *** *p* < 0.001, ^ *p* < 0.1, *α* = 0.05

Table 4 shows the multivariate regression model of personal and environmental correlates of energy drink consumption in Belgian university students. After checking for multicollinearity (showing *r* = −0.69 between perceived behavioral control and habit strength) habits strength was excluded from the multiple regression model. After controlling for age, ethnicity, parental education, and BMI, both gender and residency were found to moderate the relationship between perceived behavioral control and energy drink consumption. In male students, perceived control was negatively associated with energy drink intake (β = −0.820; *p* < 0.001); the more male students found it difficult to avoid energy drinks, the higher their energy drink consumption. In females, no relation between perceived control and energy drink intake (β = −0.212; *p* = 0.141) was detected. Although in both students living at home and students living in a student residence perceived control was negatively associated with energy drink consumption, the association was stronger in students living at home (β = −0.820; *p* < 0.001) than in students living in a student residence (β = −0.577; *p* < 0.001). Both family rules and perceived availability correlated positively with energy drink consumption; the more students were/are allowed to drink energy drinks at home, and the more students report that energy drinks are available at home/at university, the higher their energy drink intake. Gender (control variable) was negatively associated with energy drink consumption, *i.e.* female students consumed less energy drinks than their male counterparts. Perceived control was the strongest correlate of energy drink consumption (β = −0.820; *p* < 0.001). The total model explained 44.6% of the variance in energy drink consumption.

**Table 4:** Multivariate regression model of personal and environmental correlates of energy drink consumption in university students (*t*-values, β-values, Adjusted *R*^2^).

*n* = 425	*t*	β	Adj *R*^2^
*Control variables*			
Age	0.8	0.047	
Ethnicity (0 = parents from Belgian origin; 1 = one of parents from foreign origin)	0.6	0.039	
Education mother (0 = no diploma higher education; 1 = diploma higher education)	1.1	0.069	
Education father (0 = no diploma higher education; 1 = diploma higher education)	0.7	0.042	
BMI	0.7	0.045	
*Moderators*			
Gender (0 = male; 1 = female)	−2.2 *	−1.293	
Residency (0 = living at home; 1 = living in a student residence)	−0.5	−0.204	
*Personal and environmental correlates*			
Taste preference	1.1	0.078	
Attitude	−0.5	−0.040	
Individual subjective norm	−0.9	−0.086	
Perceived control	−7.5 ***	−0.820	
Self-efficacy	−0.0	0.000	
Perceived benefits	0.0	0.000	
Perceived barriers	−1.4	−0.134	
Social norm	−1.2	−0.070	
Social support	1.8 ^	0.119	
Modelling	−1.4	−0.089	
Family rules	2.2 *	0.198	
Perceived availability	2.1 *	0.203	
Distance to stores	0.9	0.053	
*Moderation effects of gender and residency*			
Individual subjective norm x gender	0.2	0.054	
Perceived control x gender	3.8 ***	1.585	
Perceived control x residency	1.9 ^	0.589	
Self-efficacy x gender	−0.2	−0.096	
Perceived benefits x residency	−1.5	−0.389	
Perceived barriers x gender	0.8	0.130	
Family rules x gender	−1.2	−0.164	
Perceived availability x gender	−1.0	−0.209	
			0.446

* *p* < 0.05, *** *p* < 0.001, ^ *p* < 0.1, *α* = 0.05.

## 4. Discussion

The first objective of this study was to assess which personal and environmental factors were related to Belgian university students’ soft drink and energy drink consumption. The second objective was to investigate whether these associations were moderated by gender or residency.

Students drank almost half a liter of soft and energy drinks per day, consisting of about 350 mL of sugar sweetened beverages per day. In contrast, students drank only 17 mL of sugar containing energy drinks per day. Soft and energy drinks combined, this corresponds to an estimated caloric value of 140 kCal/day, which is less than the 162 kCal/day reported by US students [17]. The latter suggests that Belgian students may drink less sugar sweetened beverages than their US counterparts. This difference might be explained by socio-cultural differences between the European and US beverage culture (free refills in US fast food restaurants). Due to the limited literature in this population no comparison could be made for artificially sweetened beverages or water. Importantly, in comparison to their soft and energy drink intake, Belgian students still consume a larger amount of water (650 mL) per day.

The fact that Belgian students consume about half a liter of soft and energy drinks per day corresponds with other studies detecting poor dietary habits among university students. Less than 50% of students in four European countries reported daily consumption of fruits, whereas only 15% to 32% of them reported daily vegetable consumption [37]. The same study revealed that 11% to 77% of students (depending on the country) consumed fast food at least several times per week [37]. The above suggests that European university students in general fail to meet dietary recommendations.

Individual subjective norm was the strongest correlate of soft drink consumption; the more students living at home were convinced they should minimize their soft drink consumption, the less these students consumed soft drinks. In comparison, no main effect of individual subjective norm was detected in students residing away from home. This may suggest that factors other than individual subjective norm may be more important in the beverage choice making process of students living in a student residence. For instance, previous qualitative research in university students showed that next to taste (which was not significant in the present study), price was mentioned to be one of the most important factors in choosing beverages [22,38]. Because students living in a student residence often have budgetary limitations [33], it might be that the beverage choice of students residing away from home is more subject to price than to their individual subjective norm about soft drink consumption. Future experimental studies should investigate if enhancing individual subjective norm towards avoiding soft drinks would indeed decrease students’ soft drink consumption. No relationship between individual subjective norm and energy drink consumption was detected.

Perceived behavioral control was the strongest correlate of energy drink consumption. The association between perceived behavioral control and energy drink intake was moderated by both gender and residency. The more male students found it difficult to avoid energy drinks (probably as a consequence of energy drink availability and accessibility), the higher their energy drink consumption. In females, no relationship between perceived control and energy drink intake was detected. In comparison to students living in a student residence, the association between perceived control and energy drink consumption was stronger in students living at home. Furthermore, perceived availability was positively related to energy drink intake. Because energy drink availability may play an important role with regard to (male) students’ perceived control over energy drink intake, interventions may have to focus on students’ perceived behavioral control and the physical environment at the same time. Students, but also parents and university policy makers are therefore challenged to limit respective home and campus availability of energy drinks and replace them by healthy alternatives such as water. Although not moderated by gender or residency, a similar negative relationship was detected between perceived behavioral control and soft drink consumption. Enhancing self-regulation skills such as behavioral control towards both soft and energy drink consumption may help students to make more healthful decisions and to maintain a healthy lifestyle throughout adulthood [39,40].

Although none of the previous qualitative studies mentioned self-efficacy to be influencing students’ beverage consumption, the present study did find that the more students were convinced they could avoid soft drinks in different situations, the lower their soft drink consumption. Hence, strengthening students’ self-efficacy towards avoiding soft drinks may decrease their soft drink consumption. Strategies such as self-monitoring (tracking one’s own soft drink intake), providing feedback on beverage choices, reviewing behavioral goals, providing rewards, or planning for social support, may effectively increase self-efficacy towards avoiding soft drink consumption [41]. In the present study, self-efficacy was not related to energy drink intake.

Family rules were significantly associated with both soft drink and energy drink consumption. The more male students were/are allowed (by their parents) to drink soft drinks, the higher their soft drink consumption. In comparison, no main effect of family rules were detected in female students. Regarding energy drinks, family rules were positively related to both male and female students’ energy drink intake. It has been shown that almost half (48%) of current users among university students initiated energy drink use between the ages of 16 and 20 years, whereas 42% of students were only 11 to 15 years old when consuming energy drinks for the first time [20]. Stricter family rules during (or even prior to) adolescence about soft and energy drink intake may help to decrease consumption of soft and energy drinks throughout adolescence and young adulthood, as well as in later life.

Regarding other social environmental factors, modelling was positively related to soft drink consumption in students living at home, but not in students living in a student residence. The more these students’ partner, parents, friends, or student colleagues consumed soft drinks, the higher their soft drink intake. Although recommendations from family and friends, social settings and peer pressure were mentioned as modes of initiation by Caribbean university students [20], social norm, support and modelling were not associated with energy drink consumption.

The above suggests that multilevel (combining personal with environmental) strategies may be more effective than single-level interventions. So, next to targeting psychological mechanisms, students’ (social and physical) environments should be taken into account. In other words, intervention efforts to decrease students’ soft and energy drink consumption should also try to provide a favorable environment in which students and their peers are encouraged to make healthy beverage choices. Therefore, different stakeholders (namely students, parents, but also university as well as governmental policy makers) should be involved when designing prevention strategies.

As for the unexplained variance in our analyses, it may be that students’ beverage consumption is partly determined by their past consumption habits. Although excluded from the present analyses because of multicollinearity, habit strength was previously detected to be a strong positive correlate of soft drink consumption in adolescents [42]. Furthermore, inspired by Sallis’ ecological model to explain health behavior [43], it may be that university students’ beverage choices are also influenced by other (meso and macro environmental) factors such as promotion and marketing, market regulations, and policy.

An important strength of this study is that we took possible moderators into account. About half of the significant personal and environmental correlates reported in this study were moderated by gender or residency. This indicates that intervention strategies aiming to decrease soft or energy drink consumption by targeting psychosocial constructs may not be suitable for the total group of university students. Hence, such strategies may have to approach male and female students, as well as students living at or away from home, differently.

Since behavior is dynamic in nature, a static correlational model as such has its limitations [44]. Due to its observational cross-sectional design, the present study was not able to determine causality. Therefore, future controlled trials should investigate whether changes in the above personal and environmental factors can cause changes in students’ soft and energy drink consumption. A second limitation is that we used standard portion sizes to estimate soft and energy drink consumption, as well as a self-reporting questionnaire. This may have led to an under- or overestimation of total soft and energy drink consumption. Thirdly, although it is generally recognized that some constructs derived from different psychosocial models overlap (perceived behavioral control, self-efficacy, perceived barriers), we chose to include all measures, facilitating determination of unique contributions of perceived behavioral control over self-efficacy. Because including all measures may reduce the likelihood that any of these items could emerge as significant predictors of behavior, inter-correlation analyses were run so that multicollinearity could be ruled out. Finally, all participating students were volunteers, which may have resulted in a selection bias. It might be that more healthy students participated in this study. However, sample characteristics showed sufficient variance in health related variables, such as BMI, smoking, perceived health and fitness. Unfortunately, we were not able to verify the representativeness of the assessed sample, and, therefore, we have to be cautious with generalizing results to the entire Belgian student population.

## 5. Conclusions

Interventions aiming at decreasing soft and energy drink consumption in university students should try to enhance behavioral control skills, improve individual subjective norm about beverage use, and strengthen self-efficacy towards avoiding unhealthy beverages. Moreover, these interventions should also focus on the students’ environment, by encouraging parents to implement stricter family rules about soft and energy drink intake, but also by targeting beverage use of students’ partner, parents and friends, as well as home and campus availability. When designing tailored intervention programs, male and female students as well as students living at home or living in a student residence, may have to be approached somewhat differently. Future research should investigate whether interventions focusing on the above correlates can indeed improve university students’ beverage choices.
